# Agroclimatic Evolution web application as a powerful solution for managing climate data

**DOI:** 10.1038/s41598-022-10316-7

**Published:** 2022-04-25

**Authors:** Manuel Soler-Méndez, Dolores Parras-Burgos, Rachid Benouna-Bennouna, José Miguel Molina-Martínez

**Affiliations:** 1 Agromotic Engineering and the Sea Research Group, Universidad Politécnica de Cartagena, 30202 Cartagena, Spain; 2 Department of Structures, Construction and Graphic Expression, Universidad Politécnica de Cartagena, 30202 Cartagena, Spain

**Keywords:** Climate sciences, Environmental sciences, Engineering

## Abstract

Applying the AgroClimatic Evolution web application allows inquiries being made, data being collected and variables being calculated with the data acquired from different public agrometeorological stations on a single platform. Today all these stations from Murcia and Andalusia (Spain) are included, and stations elsewhere in Spain are being incorporated. This web application also offers the possibility of including each user’s own stations, which increases the number and availability of data close to each farmer’s plots. The data collected from stations is employed to collect daily data about weather and times, which are used to calculate the reference evapotranspiration (ETo). All the data are saved in a cloud database to later consult them and study their evolution. The data provided by all the stations are validated by applying the filters indicated in Standard UNE 500540:2004 “Automatic weather stations networks” by eliminating mistaken data that could alter correct ETo calculations. With the filtered data, and having calculated ETo, the user is provided with a comparison made with the raw data supplied by public stations. The main objective of this tool is to optimize the use of water resources available from data acquisition. Managing these data will contribute to make agriculture more sustainable and compatible with the natural environment.

## Introduction

In recent decades, the agricultural sector has undergone marked transformation that has evolved over time. It can be stated that the first step began with the mechanization of farm work and the introduction of fertilizers and phytosanitary products. Later significant progress was made thanks to information and communication technologies (ICT) being introduced, which enables vast quantities of data about soil status, water availability, crop development, the amount of plant sanitary product applied to each plant, etc., to be collected and transmitted^1–6^. All this, along with historic information about weather, the progression of plagues or diseases, is leading to a major transformation in the sector that is increasing productivity and competition^7^.

Therefore, the management and integration of different databases will be one of the advances that will have a strong impact in forthcoming years because it will allow decisions to be quickly, effectively and accurately made to optimize production and costs^8^.

At the same time, this sector faces low water availability and increasingly degraded quality^9^. Therefore, efficient water resources management is the main tool to confer farms stability and sustainability^10^. In relation to this, the use of ICT is an excellent support to efficiently manage water resources^11^. Employing ICT in agriculture is based on using sensors that monitor various agri-environmental variables related to the soil–plant-atmosphere combination to quantify or estimate water requirements^9^. Knowing environmental data allows the evapotranspiration (ET) demand in an area to be calculated, with which the consumption of a given crop can be estimated by applying the corresponding corrective coefficients. Such information can help to more efficiently manage irrigation. As a network of public agrometeorological stations that obtain such data exists, placing them at farmers’ disposal seems important.

Different websites can be found on the Internet that offer interesting agroclimate information to be consulted^12–14^, although some shortcomings have been detected that should be stressed. Some examples of these shortcomings are: (i) the web format does not match that of mobile devices; (ii) data consultations are awkward and slow; (iii) consulting time data is impossible; (iv) forms to consult data are lacking; (v) data cannot be plotted as graphs; (vi) there are no weather forecasts; (vii) no basic data about stations to make inquiries with are available.

In order to overcome these deficiencies and to offer users a practical and efficient tool, a web application has been developed to: (i) consult ETo; (ii) consult data from the different selected stations; (iii) obtain weather forecasts. The aim of this project is to have a web application that meets a series of minimum characteristics that: (a) is simple; (b) offers a responsive presentation, that is, it adapts to different devices; (c) serves to collect data in real time to calculate daily ET and its time to be later stored in a database.

Another objective of this project is for users to make accurate irrigation decisions by bearing in mind information on the ET demand offered by this tool. This allows improved water resources management without having to invest in any sensors network.

## Materials and methods

### Websites analysis

There are two types of web-based weather information systems:

- Private stations: These allow data to be obtained close to each farmer's plot, provided that as many weather stations as are of interest are invested. This is an advantage in terms of the representativeness of the data for each farmer; however, it has the disadvantage of the high cost of the investment.
  i. An example is the service offered by Sencrop (www.sencrop.com), which centralises all the data from privately installed stations on one platform (if the owner so wishes) and makes them available to other users, thus reducing the investment cost.
  ii. Another example is offered by Envira IOT (www.enviraiot.es/monitorizacion-meteorologica-agricultura-precision/), which installs a private weather station and offers data provided with algorithms that facilitate user interpretation.
- Public stations: There are numerous web services that offer historical weather data collected from the existing network of weather stations in Spain. In general, access to these data is free of charge, but the microclimate of a particular farm may differ from the data provided by the nearest weather station.
  i. SIAR (Agro-climatic Information System for Irrigation) (https://eportal.mapa.gob.es/websiar/Inicio.aspx) offers data from stations located throughout Spain, through its website (and even has a mobile App).
  ii. At the level of autonomous communities, some offer this service through their own portals, with data from the same stations: SIAM of the Region of Murcia (www.siam.imida.es), InfoRiego of the “Junta de Castilla y León” (www.inforiego.org/opencms/opencms/info_meteo/index.html), RIA of the “Junta de Andalucía” (https://www.juntadeandalucia.es/agriculturaypesca/ifapa/riaweb/web/), among others.

The potential of the data collected in these services lies in their free access, and in the quality of the data coming from a Spanish network. On the other hand, the consultation of data is, in general, not user-friendly, nor is it adapted to mobile devices. In addition, data are consulted whose processing requires considerable office skills to be interpreted. These services lack the ability to graph the data, and the possibility of easily storing them in their own database.

Taking into account the study carried out, the AgroClimatic Evolution web application was developed, designed to provide free access to data to users, in a user-friendly and simple way. But this application is part of a broader project, the GENHIDRO platform (www.genhidro.es), aimed at the efficient and autonomous management of fertigation systems.

Therefore, the aim of this project is to ensure that the data obtained through AgroClimatic Evolution are not merely a mere visualisation and storage of data, but feed a more complex decision-making system for advice and even the direct management of fertigation systems. GENHIDRO is a platform under continuous development and aims to offer the user the possibility of interacting with the irrigation system based on data collected, among others, from meteorological information services such as those consulted through AgroClimatic Evolution.

### Theorical background

The ETo was calculated in this project. This ETo concept occurs under certain conditions, on a reference surface and with no water restrictions. The ETo is a variable to study atmospheric ET demand regardless of crop type and crop development.

By relating ET to a given surface, a reference ET is obtained. From this value, ET values on other surfaces can be related. This will allow analyses based on ET alone, and independent of other site-specific variables. Thus, ETo will be the reference ET under certain growing conditions, for a reference crop. The ETo value will express the evapotranspiration capacity of the atmosphere at a given location and time of year; but it does not take into account the characteristics of the crop or soil type. The great advantage of using ETo to estimate water requirements is that its value only depends on climatic parameters, which are easy to obtain.

To understand the ETo concept, it is necessary to define what a reference surface is. According to FAO-56, it is a “hypothetical reference crop with crop height of 0.12 m, a fixed surface resistance of 70 s m^-1^ and an albedo value (i.e., portion of light reflected by the leaf surface) of 0.23”^15^.

To calculate this parameter, the FAO Penman–Monteith method is recommended because it is the only standardized method that determines ETo with climate parameters^16^. This method of Zotarelli was selected because it roughly approaches the ETo of any town, has robust physical bases, and explicitly incorporates physiological and aerodynamic parameters. The data required to apply the FAO Penman–Monteith method are location, temperature, relative humidity, radiation and wind.

Determining ETo is extremely important for estimating a crop’s water requirements or for conducting studies regardless of the crop type to be grown. Knowledge about ETo and the grown crop allows adjusted irrigation doses to be established and crop performance to improve.

To calculate the ETo, we applied the equations of the FAO Penman–Monteith method available in FAO-56. We began with daily ETo, calculated by the expression below:

1 $$ET_{o} = \frac{{0.408 \Delta \left( {R_{n} - G} \right) + \gamma \frac{900}{{T + 273}} u_{2} \left( {e_{s} - e_{a} } \right)}}{{\Delta + \gamma \left( {1 + 0.3 u_{2} } \right)}}$$

where:

*ET*_*o *_ Reference evapotranspiration [mm day^-1^]

*R*_*n*_Net radiation the crop’s surface(MJ m^-2^day^-1^)

*Ra* Extraterrestial radiation (mm day^-1^)

*G* Ground heat flow(MJ m^-2^day^-1^)

*T * Mean air temperature at a heigjt of 2 m (°C)

*U*_*2*_ Wind speed at a height of 2m (m s^-1^)

*E*_*s*_  Vapour saturation pressure (kPa)

*e*_*a*_  Real vapor pressure (kPa)

*e*_*s*_*-e*_*a*_ No vapor pressure (kPa)

$\Delta$ Vapor pressure curve slope (kPa °C^-1^)

$\gamma$ Psychometric constant (kPa °C^-1^)

For the calculation of each and every one of the parameters involved in Eq. (1), the methodology established by Zotarelli et al.^16^ has been followed. Thus, the daily ETo estimate is obtained, expressed in mm·day^-1^.

To obtain the ETo value at the hourly level, the expressions indicated by Zotarelli et al.^16^ vary slightly. Thus, for hourly periods, the FAO Penman–Monteith equation for the calculation of ET is modified as follows:

2 $$ET_{o} = \frac{{0.408 \Delta \left( {R_{n} - G} \right) + \gamma \frac{37}{{T_{hr} + 273}} u_{2} \left( {e^{o} \left( {T_{hr} } \right) - e_{a} } \right)}}{{\Delta + \gamma \left( {1 + 0.24 u_{2} } \right)}}$$

where:

*ET*_*o*_  Reference evapotranspiration [mm hour^-1^]

*R*_*n*_ Net radiation on the reference surface [MJ m^-2^ hour^-1^]

*G * Ground heat flow density [MJ m^-2^ hour^-1^]

*T*_*hr*_ Mean air temperature every hour [°C]

$\Delta$ Vapor saturation pressure curve slope in Thr [kPaC^-1^] and psychometric constant[kPa °C^-1^]

*e (T*_*hr*_*)* Vapor saturation pressure at *T*_*hr*_

*e*_*a*_ Average real vapor pressure times [kPa]

*U*_*2*_ Average wind spee times [m s^-1^]

The adjustments made to the equations proposed by Zoratelli et al.^16^, in order to obtain the hourly ETo values, are shown below:

Real vapor pressure (*e*_*a*_) is calculated as:

3 $$e_{a} = e^{o} \left( {T_{hr} } \right)\frac{{HR_{hr} }}{100}$$

where:

*e*_*a*_ Average real vapor pressure times [kPa]

*e (T*_*hr*_*)* Vapor saturation pressure at *T*_*hr*_

*HR*_*hr*_ Average relative humidity time [%]

The net radiation calculation varies for time periods; first, the expression to calculate extraterrestrial radiation (R_a_) becomes:

4 $$R_{a} = \frac{24 \cdot 60}{{\uppi }} G_{sc} d_{r} \left[ {\left( {w_{2} - w_{1} } \right)\sin \left( \varphi \right)\sin \left( \delta \right) + \cos \left( \varphi \right)\cos \left( \delta \right){\text{sin}}\left( {w_{1} } \right)} \right]$$

where:

*R*_*a*_ Extraterrestial radiation per hour [MJ m^-2^ hour^-1^]

*G*_*sc*_ Solar constant =0.082 MJ m^-2^ min^1^

*d*_*r*_ Realative inverse Earth −  Sun distance

$\delta$ Solar declination [rad]

$$\phi$$ Latitude [rad]

*W*_1_ Radiation angle at the beginning of the period [rad]

*W*_2_ Radiation angle at the end of the period [rad]

The initial and final radiation angles are given by:

5 $$w_{1} = w - \frac{{\pi t_{1} }}{24}$$

6 $$w_{2} = w + \frac{{\pi t_{1} }}{24}$$

where:

*w* Sun angle when the midpoint of the considered period is reached [rad]

*t*_1_ Duration of the considered period, 1 for time periods

The procedure followed to calculate net radiation is the same as that for daily period, except for Eqs. 4, 5 and 6.

In this case, heat flux is not longer negligible. The G value, according to FAO-56^15^, can be reached during light periods by:

7 $$G_{hr} = 0.1{ }R_{n} { }$$

and for night periods:

8 $$G_{hr} = 0.5{ }R_{n} { }$$

With these changes in the expressions, the hourly ETo can be properly estimated. As it is now known which calculation procedure is to be used, it is possible to study how the web application works.

### Web application

This web application can be used with the link that follows: (http://josemiguel.myqnapcloud.com:49169/AgroClimatic-Evolution/).

**Figure 1:**
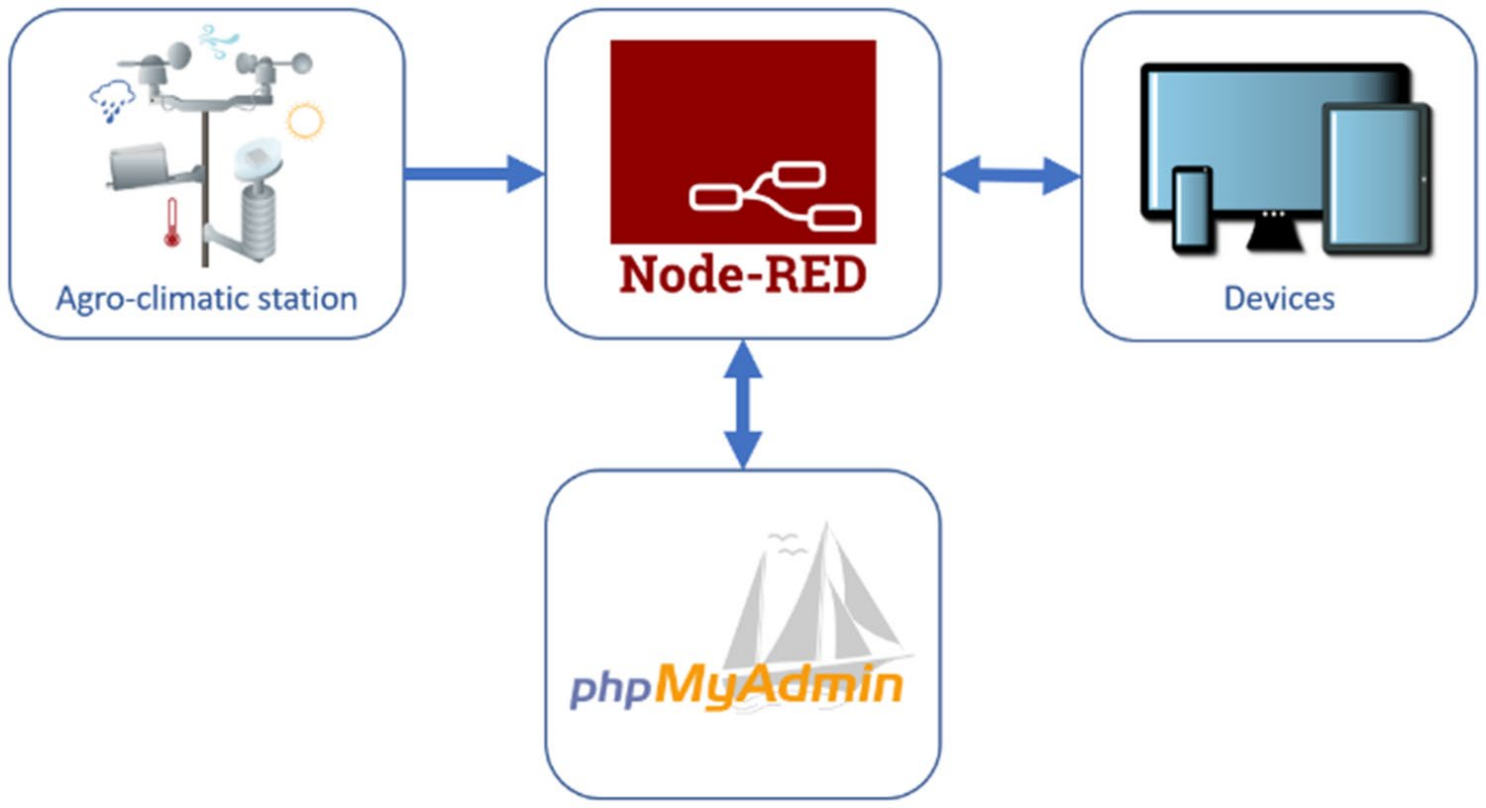
Outline of how the AgroClimatic Evolution tool operates.

The AgroClimatic Evolution (v1.0), web application can be used in a PC or a mobile device (Android or IOS). As this web application is executed by means of a server, a web browser is necessary, and Chrome is recommended. This tools’ operation is outlined in the figure below (Fig. 1).

Node-RED (https://nodered.org/) is the core of the web application's operation. It is a programming tool designed to communicate with different hardware, simplifying the processes of sending and receiving information as much as possible. It allows to connect different hardware devices, use APIs to perform communications, and connect with other services, in a very innovative and interesting way. The programming is visual, based on flows, so it does not require a very advanced level of programming.

Node-RED uses the "*http request*" node to request data from a specific weather station to the corresponding meteorological service. The data collected are: weather station, date, temperature, relative humidity, radiation, wind speed, wind direction and dew point temperature. This information is stored in the database, and phpMyAdmin (https://www.phpmyadmin.net/), which is a free open-source software tool, is used to manage and administer the database. Another Node-RED node is in charge of calculating the hourly or daily ETo values from the obtained meteorological data. This information is stored in the database. With the "*uibuilder*" node, the user interface interprets the information request made by the user, and extracts the appropriate information from the database to display it on the interface. This whole process works on the back-end.

The system is connected to different APIs and/or web services to acquire interesting weather data: (i) the API that OpenWeather (https://openweathermap.org/) provides for weather forecasts; (ii) the API that the “Junta de Andalucía” (the Regional Government of Andalusia, Spain) provides for weather data; (iii) obtaining data from websites of SIAM (the Farming Information System of Murcia) and SIAR (the Agroclimate Information System for Irrigation) by scraping every 6 min (Table 1). The public stations used respect the WMO standards (World Meteorological Organization, https://community.wmo.int/standards-and-requirements-climate-observations), and its main characteristics of equipment and data management is described by María del Carmen Caro Vela from SIAR ^17^.

**Table 1:** Access to different APIs.

OpenWeather API call format	https://api.openweathermap.org/data/2.5/onecall?lat={lat}lon={lon}exclude={part}appid={APIkey}
API Junta de Andalucía call format	https://www.juntadeandalucia.es/agriculturaypesca/ifapa/riaws/datosdiarios/forceEt0/{codigoProvincia}/{codigoEstacion}/{fhInicio}/{fhFin}}
SIAM and SIAR data acquisition	By scraping

The data provided by all the stations are validated by applying filters at levels 0 and 1 as indicated in Standard UNE 500540:2004 “Automatic weather stations networks: Guidance for the validation of the weather data from the station networks. Real time validation”. At level 0, the structure of the data record is validated, i.e. that the number of data received is the same as the number of data expected, as well as the date and/or time, taking as invalid those that do not comply. Level 1 establishes the physical limits within which the different climate variables must move, beyond which data are considered null. Table 2 shows the physical and instrumental limits established by the UNE 500540:2004 standard. With the filtered data, and once the ETo calculations have been obtained, the user is provided with a comparison made with the raw data supplied by the public stations.

**Table 2:** Physical limits of the different weather variables (UNE 500540:2004).

Weather variable	Unit	Range
Air temperature	°C	− 35/55
Relative humidity in air	%	0/100
Wind speed	m·s^−1^	0/75
Direction wind blows in	Degrees	0/360
Pressure	hPa	700/1080
Global solar radiation	W·m^−2^	− 1/1400
Precipitation in 10 min	mm	0/50

To validate the correct execution of the process of obtaining data from the meteorological services mentioned, the following test was carried out: a weather station was taken at random, during a randomly chosen month, and the data provided directly by the service were compared with those generated by the web application developed (Tables 3, 4, and 5).

**Table 3:** Comparison of temperature data obtained by the AgroClimatic web application and by the Meteorological Data Service.

Date	Max. temperature (°C)			Min. temperature (°C)			Mean temperature (°C)		
	WSD	ACED	Error	WSD	ACED	Error	WSD	ACED	Error
04/10/2021	25.40	25.44	0%	17.30	17.26	0%	20.50	20.51	0%
05/10/2021	24.00	24.03	0%	15.90	15.87	0%	19.70	19.66	0%
06/10/2021	25.70	25.69	0%	15.30	15.26	0%	20.90	20.89	0%
07/10/2021	26.40	26.37	0%	17.50	17.52	0%	21.10	21.07	0%
08/10/2021	24.90	24.90	0%	17.70	17.66	0%	21.20	21.22	0%
09/10/2021	2500	24.96	0%	18.80	18.79	0%	21.50	21.47	0%
10/10/2021	25.90	25.90	0%	17.20	17.19	0%	21.30	21.33	0%
11/10/2021	27.10	27.10	0%	17.40	17.39	0%	21.90	21.87	0%
12/10/2021	25.60	25.63	0%	17.70	17.66	0%	21.10	21.10	0%
13/10/2021	24.20	24.17	0%	16.70	16.66	0%	19.70	19.67	0%
14/10/2021	24.40	24.43	0%	16.60	16.60	0%	20.10	20.07	0%
15/10/2021	24.70	24.70	0%	15.90	15.87	0%	19.60	19.58	0%
16/10/2021	23.30	23.30	0%	15.90	15.93	0%	19.50	19.54	0%
17/10/2021	24.80	24.84	0%	18.50	18.46	0%	20.80	20.77	0%
18/10/2021	25.00	24.97	0%	17.50	17.46	0%	20.60	20.59	0%
19/10/2021	26.00	25.97	0%	16.90	16.86	0%	20.90	20.90	0%
20/10/2021	24.40	24.43	0%	16.60	16.59	0%	20.30	20.32	0%
21/10/2021	22.60	22.63	0%	15.10	15.06	0%	18.70	18.69	0%
22/10/2021	23.90	23.90	0%	16.10	16.06	0%	19.40	19.37	0%
23/10/2021	23.70	23.70	0%	16.10	16.06	0%	19.40	19.37	0%
24/10/2021	23.40	23.38	0%	15.10	15.13	0%	18.50	18.51	0%
25/10/2021	22.60	22.63	0%	14.90	14.93	0%	18.40	18.37	0%
26/10/2021	24.00	24.04	0%	16.50	16.47	0%	19.50	19.45	0%
27/10/2021	24.60	24.63	0%	16.30	16.33	0%	19.40	19.42	0%
28/10/2021	22.80	22.76	0%	14.90	14.93	0%	17.90	17.93	0%
29/10/2021	21.30	21.25	0%	14.90	14.93	0%	18.40	18.40	0%
30/10/2021	22.60	22.64	0%	19.30	19.32	0%	20.30	20.34	0%
31/10/2021	21.10	21.11	0%	18.40	18.39	0%	19.80	19.84	0%

WSD: Weather Service Data.

ACED: AgroClimatic Evolution Data.

**Table 4:** Comparison for relative humidity and solar radiation data obtained by the AgroClimatic web application and by the Meteorological Data Service.

Date	Max. RH (%)			Min. RH (%)			Mean SR (W/m^2^)		
	WSD	ACED	Error (%)	WSD	ACED	Error (%)	WSD	ACED	Error (%)
04/10/2021	63.60	63.60	0	31.00	30.96	0	14.40	14.41	0
05/10/2021	72.30	72.30	0	41.10	41.11	0	18.90	18.90	0
06/10/2021	81.10	81.10	0	34.30	34.31	0	19.10	19.09	0
07/10/2021	81.80	81.80	0	48.60	48.56	0	18.50	18.54	0
08/10/2021	84.50	84.50	0	56.70	56.65	0	18.10	18.12	0
09/10/2021	85.00	85.00	0	56.20	56.22	0	16.10	16.05	0
10/10/2021	80.50	80.50	0	47.60	47.62	0	18.00	17.96	0
11/10/2021	70.30	70.30	0	37.20	37.22	0	16.20	16.21	0
12/10/2021	81.90	81.90	0	48.30	48.29	0	15.00	14.96	0
13/10/2021	83.10	83.10	0	52.20	52.23	0	13.60	13.55	0
14/10/2021	82.60	82.60	0	48.60	48.62	0	17.40	17.44	0
15/10/2021	77.50	77.50	0	38.80	38.76	0	16.90	16.87	0
16/10/2021	90.00	90.00	0	45.00	44.99	0	16.20	16.23	0
17/10/2021	93.40	93.40	0	54.00	53.97	0	15.70	15.68	0
18/10/2021	91.60	91.60	0	44.00	44.03	0	15.80	15.77	0
19/10/2021	78.40	78.40	0	43.00	43.02	0	16.30	16.32	0
20/10/2021	82.90	82.90	0	29.80	29.76	0	16.50	16.48	0
21/10/2021	94.90	94.90	0	41.20	41.18	0	15.60	15.64	0
22/10/2021	91.00	91.00	0	52.70	52.71	0	14.90	14.90	0
23/10/2021	80.10	80.10	0	39.70	39.70	0	14.70	14.73	0
24/10/2021	81.40	81.40	0	44.80	44.84	0	15.90	15.88	0
25/10/2021	82.70	82.70	0	50.50	50.45	0	13.90	13.86	0
26/10/2021	79.40	79.40	0	30.90	30.89	0	12.90	12.90	0
27/10/2021	75.70	75.70	0	43.10	43.11	0	12.90	12.86	0
28/10/2021	81.80	81.80	0	43.60	43.57	0	14.70	14.65	0
29/10/2021	82.70	82.70	0	50.70	50.65	0	11.00	11.02	0
30/10/2021	87.70	87.70	0	57.50	57.51	0	10.00	10.03	0
31/10/2021	99.90	99.90	0	74.30	74.30	0	13.30	13.32	0

RH: Relative Humidity.

SR: Solar Radiation.

**Table 5:** Comparison for wind speed and ETo data obtained by the AgroClimatic web application and by the Meteorological Data Service.

Date	Mean WS (m/s)			ETo (mm/día)		
	WSD	ACED	Error (%)	WSD	ACED	Error (%)
04/10/2021	1.10	1.11	1	3.1	3.14	1
05/10/2021	1.00	1.01	1	3.1	3.14	1
06/10/2021	0.60	0.62	3	2.9	2.93	1
07/10/2021	0.90	0.93	3	3.1	3.15	2
08/10/2021	0.90	0.91	1	2.9	2.95	2
09/10/2021	0.80	0.77	− 4	2.7	2.70	0
10/10/2021	1.40	1.45	4	3.3	3.33	1
11/10/2021	1.60	1.61	1	3.6	3.60	0
12/10/2021	1.10	1.06	− 4	2.8	2.78	− 1
13/10/2021	0.80	0.79	− 1	2.3	2.35	2
14/10/2021	1.00	1.01	1	2.8	2.82	1
15/10/2021	1.10	1.12	2	2.9	2.93	1
16/10/2021	1.10	1.06	− 4	2.6	2.63	1
17/10/2021	0.60	0.64	7	2.4	2.44	2
18/10/2021	1.10	1.13	3	2.7	2.74	1
19/10/2021	1.20	1.20	0	2.9	2.94	1
20/10/2021	1.50	1.49	− 1	3.1	3.13	1
21/10/2021	1.10	1.09	− 1	2.4	2.47	3
22/10/2021	1.20	1.17	− 3	2.4	2.45	2
23/10/2021	1.20	1.19	− 1	2.6	2.61	0
24/10/2021	1.20	1.24	3	2.6	2.61	0
25/10/2021	1.20	1.17	− 3	2.3	2.31	0
26/10/2021	1.30	1.27	-2	2.6	2.65	2
27/10/2021	1.30	1.33	2	2.6	2.61	0
28/10/2021	1.00	1.02	2	2.3	2.29	0
29/10/2021	2.00	2.00	0	2.4	2.39	0
30/10/2021	5.10	5.09	0	3.2	3.21	0
31/10/2021	5.00	5.04	1	2.1	2.15	2

WS: Wind Speed.

To achieve an attractive and practical front-end, the information has been organised with HTML, CSS and JavaScript programming.

## Results

**Figure 2:**
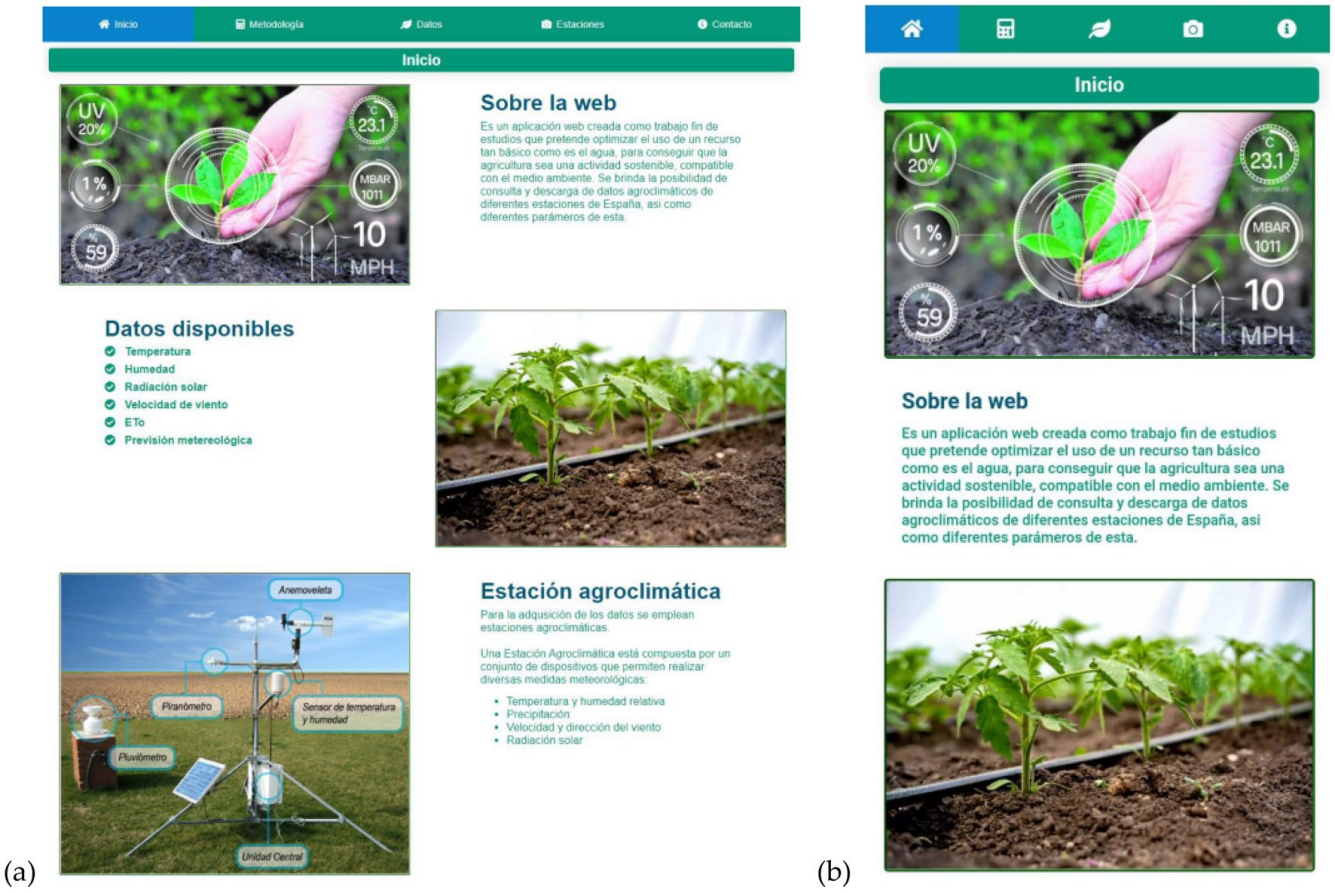
View of the initial tab in (**a**) the web application and (**b**) the mobile device.

**Figure 3:**
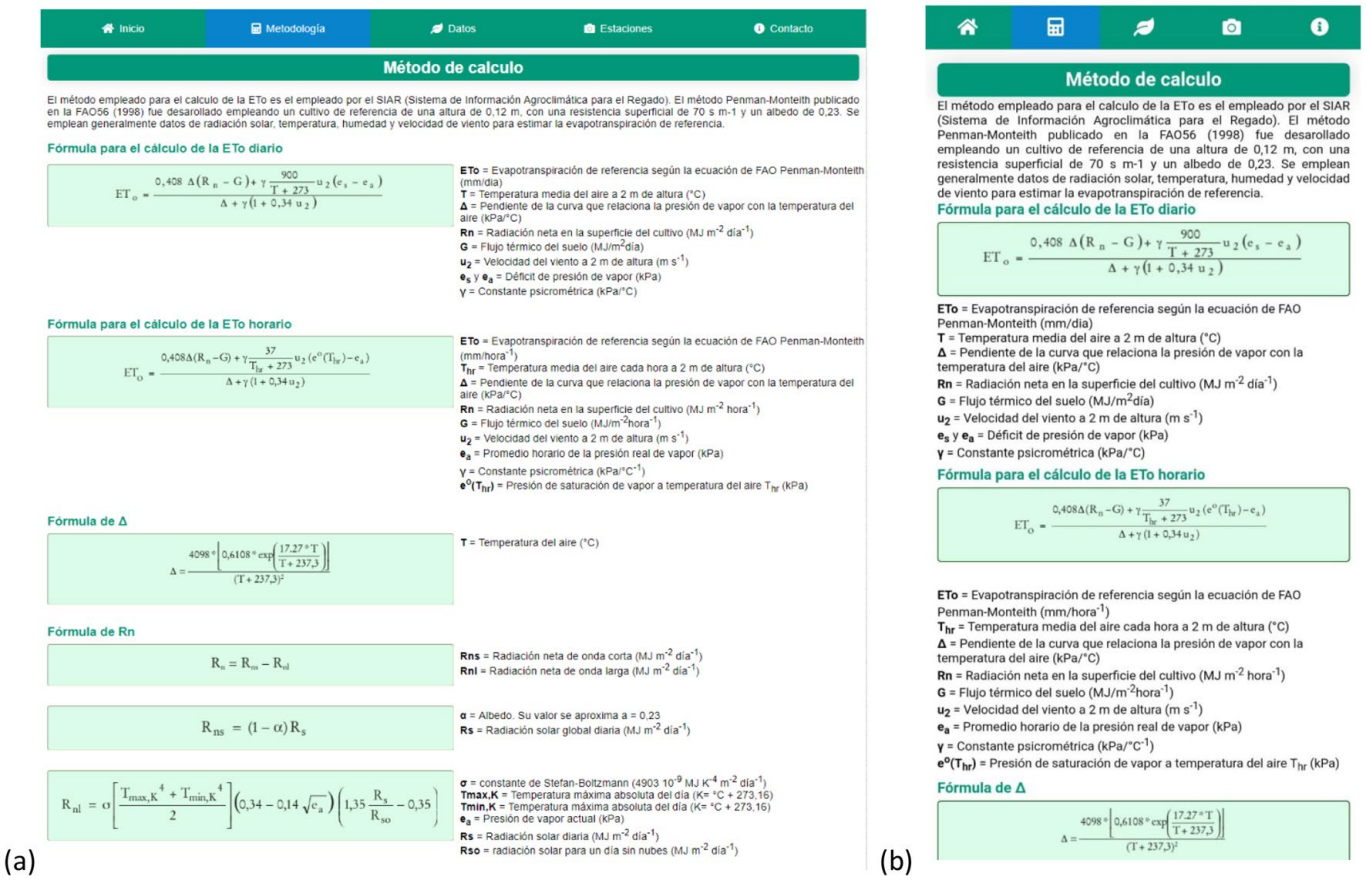
View of the tab with information about the methodology followed in (**a**) the web application and (**b**) the mobile device.

The result of this work is a user interface which shows the information that users require and request. This information is taken from databases and the different APIs. The application’s interface is divided into different tabs as the following screenshots show. The Home Page offers general information about the tool (Fig. 2). The second tab shows the followed methodology described in the previous section (Fig. 3).

**Figure 4:**
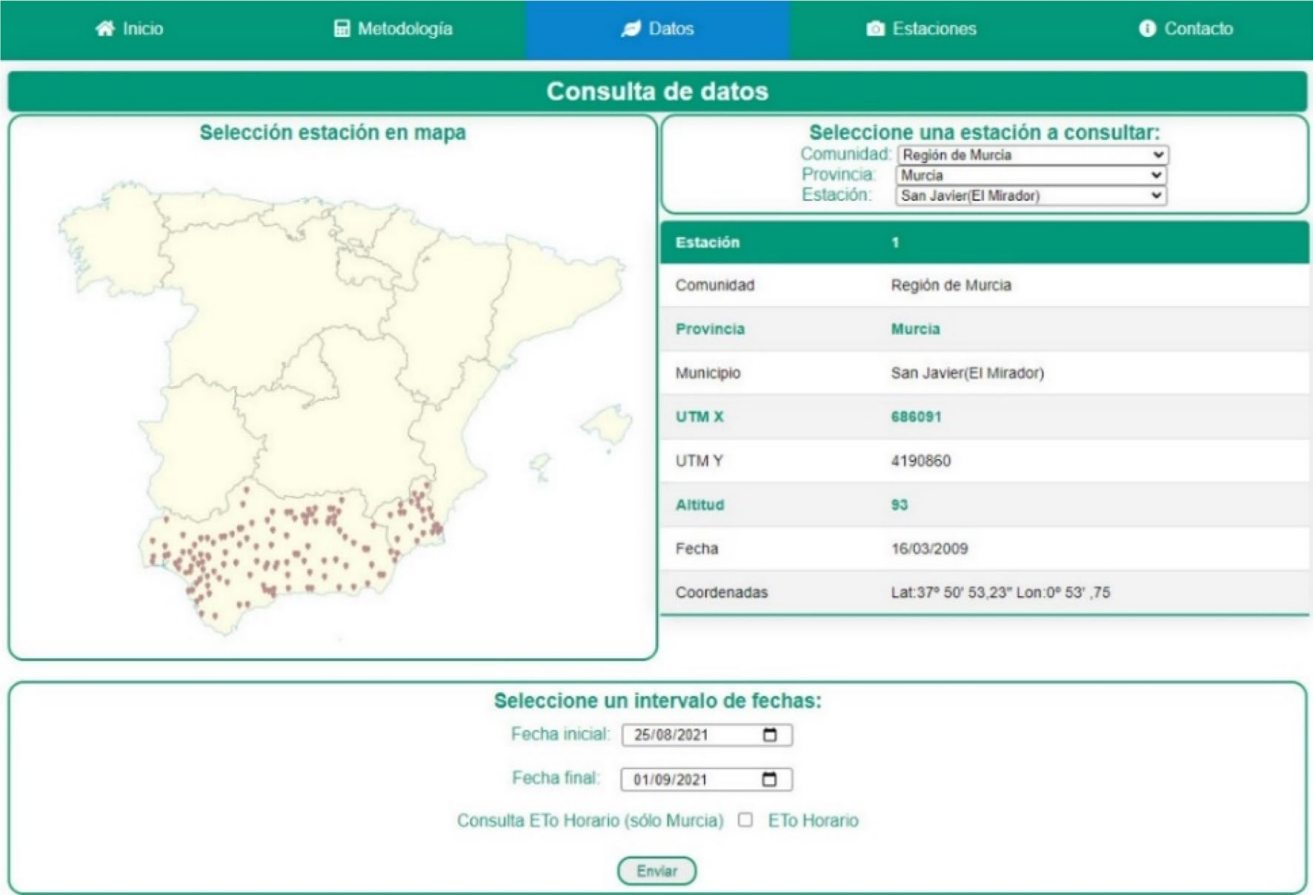
View of the tab to access the area where the data on the web can be consulted.

**Figure 5:**
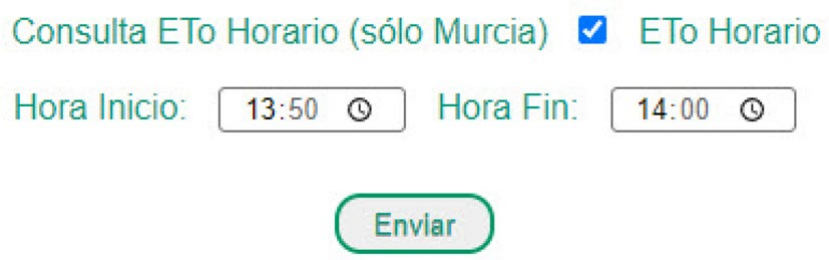
View of the hourly interval selection.

The third tab is Data, where various available parameters can be consulted (Fig. 4). Here the desired agrometeorological station can be selected by the interactive map on the left or by unfolding the menus on the right. After selecting a station, a table appears with basic and simplified information to learn the general details of what is selected. The map that opens was developed by a scalable vector graphics (SVG) editor. The advantage of using SVG is the scalability with which adjustments can be made in different sizes with-out losing properties. Different sections appear at the bottom of the screen to select the desired interval of dates and, for the Murcia Region (Spain), the option to consult the hourly ETo appears (Fig. 5).

**Figure 6:**
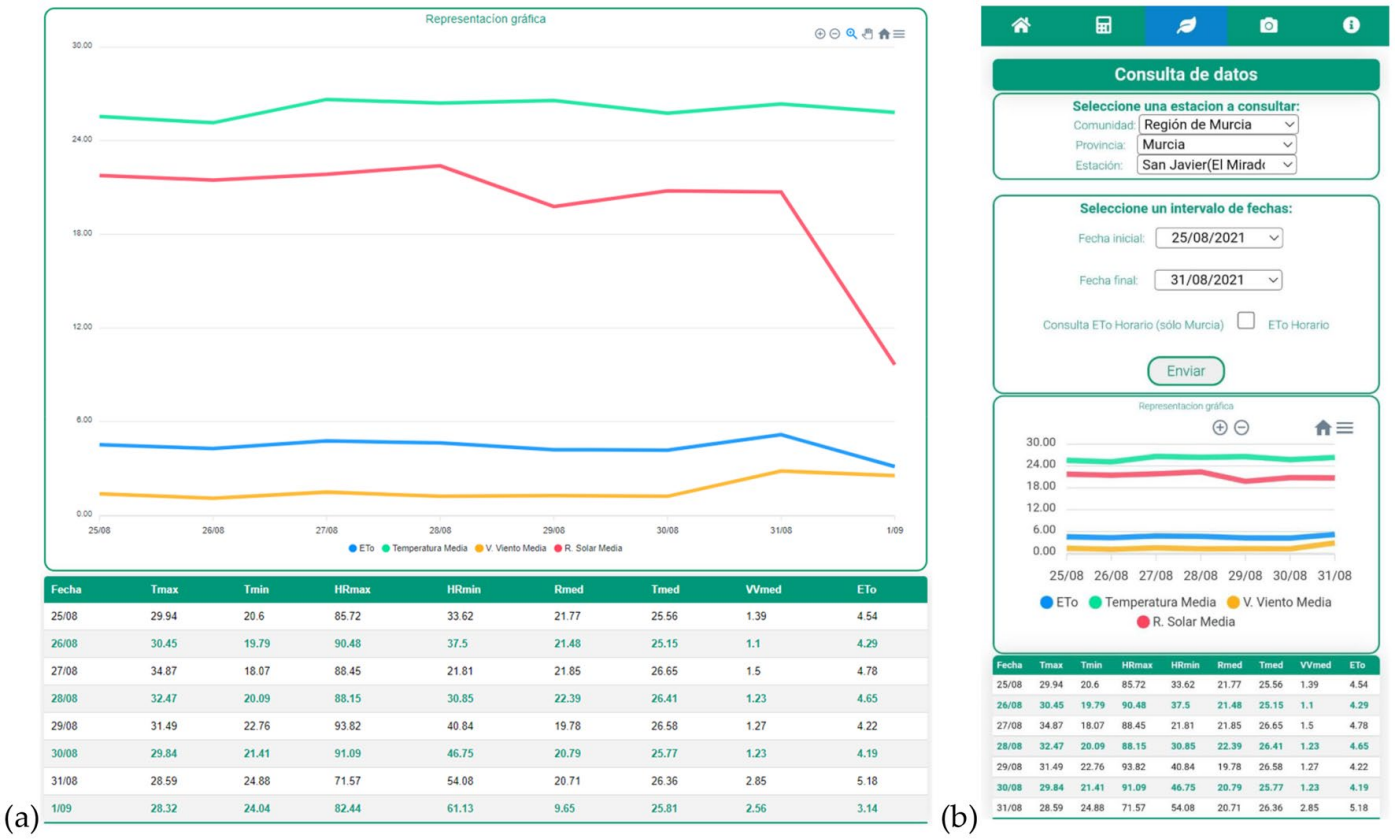
View of the Data tab as a graph and table in (**a**) the web application and (**b**) the mobile device.

**Figure 7:**
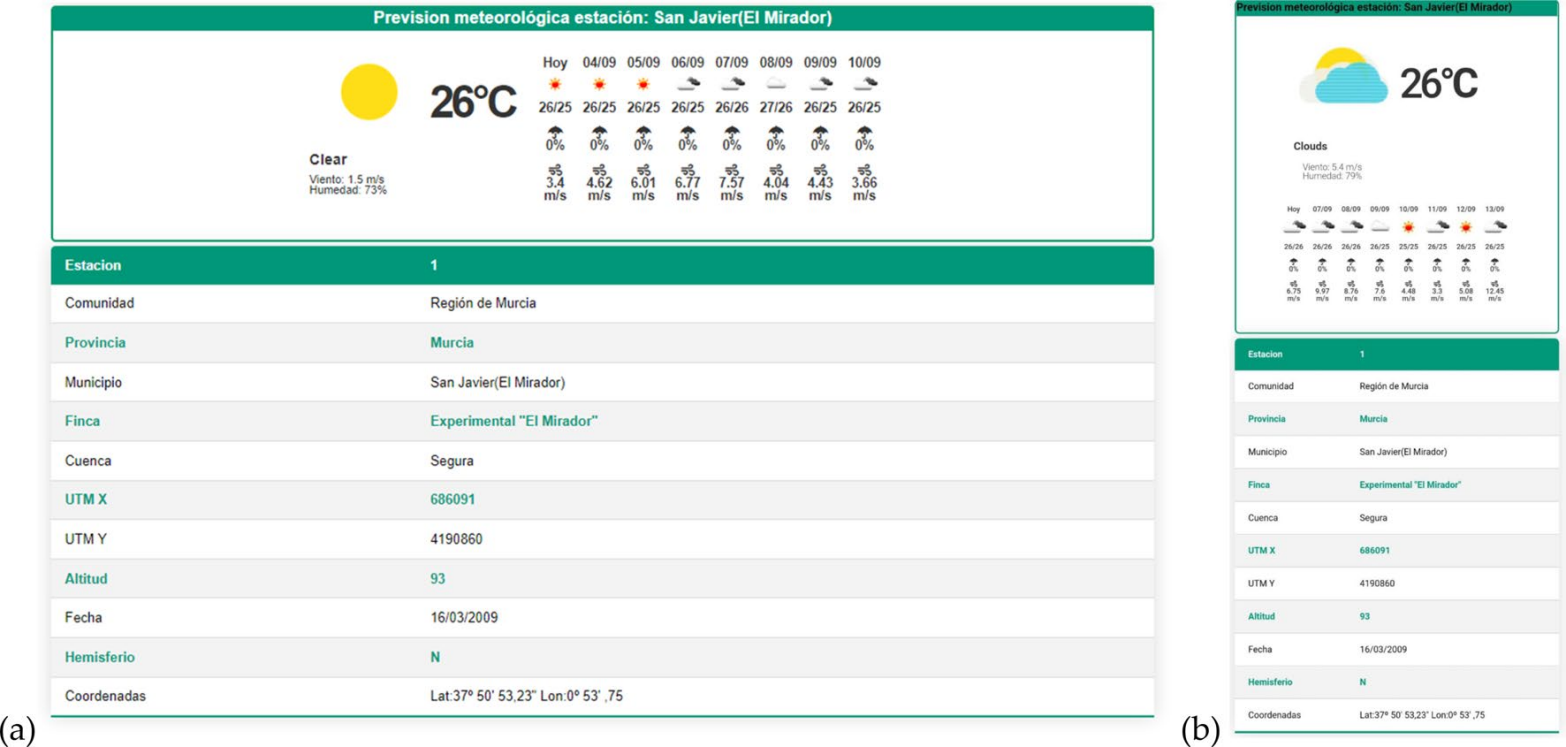
View of the weather forecast and data from the selected station in the (**a**) web application and (**b**) the mobile device.

Having selected the agrometeorological station and the desired intervals, a figure and a table appear with the requested data (Fig. 6). Moreover, the Data page also shows the weather forecast, plus further data from the selected station (Fig. 7).

**Figure 8:**
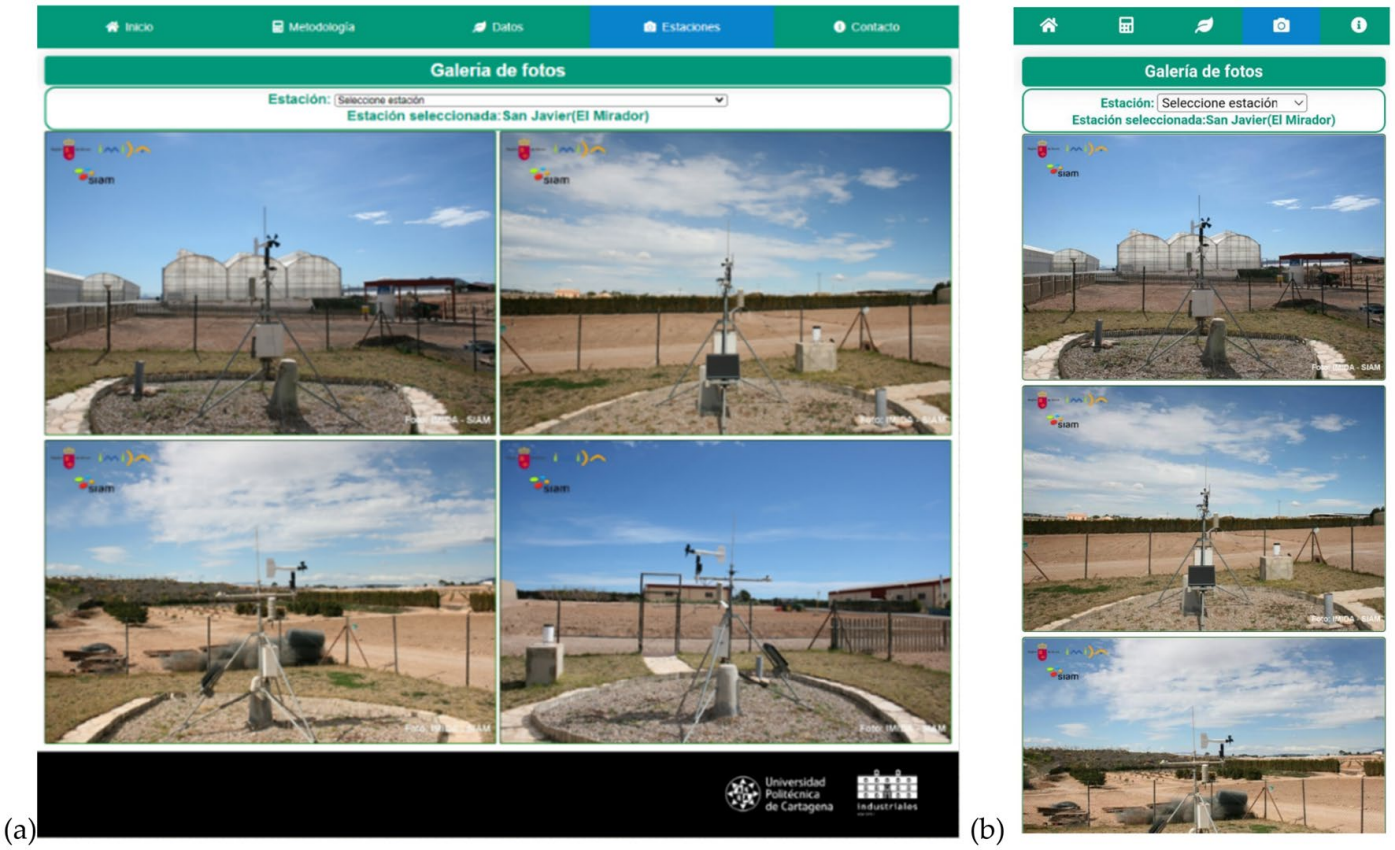
View of the Stations page in (**a**) the web application and (**b**) the mobile device.

The next tab of the web application is that of stations. It shows a picture gallery to know agrometeorological stations’ location (Fig. 8). An unfolding menu appears at the top with all the options of the available stations. When a station is selected, the related pictures are automatically obtained. The images available in the Murcia Region belong to the SIAM ^13^. The last tab that the web application offers is the Contact tab, with information about location, social networks and contact details. Finally, the web application is responsive and adapts to different devices.

With this study, what has been achieved is to unify valuable information for the farmer, in a responsive web tool, with user-friendly visualisation on both PC and mobile devices. It also allows the data to be integrated into the GENHIDRO platform, from which the fertigation system can be controlled, thus facilitating the farmer's work. In current solutions, either a costly tool is available through private weather stations, or an obsolete public data tool is available, but which in no case allows interaction with the fertigation system.

## Conclusions

The AgroClimatic Evolution web application considers developing irrigation management software with data collected from agrometeorological stations’ sensors provided or owned by different organizations. With the data offered by agrometeorological stations, an adaptive and responsive web application was developed for different kinds of devices that allows daily and hourly ET to be consulted for distinct stations. The intention of this tool is to improve irrigation management by making better use of water and suitably meeting crop needs by collecting and analyzing these data.

This can be achieved as follows: (i) the tool facilitates calculating the climate’s ET demand; (ii) by knowing crop coefficients, crop water requirements are estimated; (iii) by knowing crop irrigation needs, both the quantities of supplied water and CO_2_ flow rates from land can be reduced, which also helps to reduce the carbon footprint. By doing so, and following the agricultural policy proposals of the Council Commission and the European Parliament about the European Green Pact, whose aim is to pursue a climatically neutral Europe by 2050, work will be done to help to fulfill this objective. With the developed web application, access can be gained to all information from any device in a much simpler and more practical way to consult ETo, weather forecasts and details about the selected stations.
